# Effect of adjuvant chemotherapy on survival benefit in stage III colon cancer patients stratified by age: a Japanese real-world cohort study

**DOI:** 10.1186/s12885-019-6508-1

**Published:** 2020-01-06

**Authors:** Hidetaka Kawamura, Toshitaka Morishima, Akira Sato, Michitaka Honda, Isao Miyashiro

**Affiliations:** 1 Cancer Control Center, Osaka International Cancer Institute, 3-1-69 Otemae, Chuo-ku, Osaka, 541-8567 Japan; 2 Department of Minimally Invasive Surgical and Medical Oncology, Fukushima Medical University, Fukushima, Japan

**Keywords:** Adjuvant chemotherapy, Administrative claims, Colonic neoplasms, Health services for the aged, Medical record linkage, Multicenter study, Observational study, Propensity score, Registries, Survival analysis

## Abstract

**Background:**

Adjuvant chemotherapy is relatively underused in older patients with colon cancer in Japan, and its age-specific effects on clinical outcomes remain unclear. This study aimed to assess the effect of adjuvant chemotherapy on survival benefit in stage III colon cancer patients stratified by age in a Japanese real-world setting.

**Methods:**

In this multi-center retrospective cohort study, we analyzed patient-level information through a record linkage of population-based cancer registry data and administrative claims data. The study population comprised patients aged ≥18 years who received a pathological diagnosis of stage III colon cancer and underwent curative resection between 2010 and 2014 at 36 cancer care hospitals in Osaka Prefecture, Japan. Patients were divided into two groups based on age at diagnosis (< 75 and ≥ 75 years). The effect of adjuvant chemotherapy was analyzed using Cox proportional hazards regression models for all-cause mortality with inverse probability weighting of propensity scores. Adjusted hazard ratios were estimated for both age groups.

**Results:**

A total of 783 patients were analyzed; 476 (60.8%) were aged < 75 years and 307 (39.2%) were aged ≥75 years. The proportion of older patients who received adjuvant chemotherapy (36.8%) was substantially lower than that of younger patients (73.3%). In addition, the effect of adjuvant chemotherapy was different between the age groups: the adjusted hazard ratio was 0.56 (95% confidence interval: 0.33–0.94, *P* = 0.027) in younger patients and 1.07 (0.66–1.74, *P* = 0.78) in older patients.

**Conclusions:**

The clinical effectiveness of adjuvant chemotherapy in older patients with stage III colon cancer appears limited under current utilization practices.

## Background

Colon cancer is one of the most common cancers worldwide, and its incidence is increasing in the older population [1–3]. Older adults with colon cancer are generally more susceptible to issues such as impaired organ function, comorbidities, and polypharmacy [4, 5]. These factors can contribute to a higher frequency and severity of adverse events in older patients undergoing medical treatment [6, 7].

Approximately one-fourth of incident colon cancer patients present with node-positive stage III disease [8]. Stage III colon cancer patients have a high risk of recurrence after undergoing curative resection, [2, 3] and several randomized controlled trials (RCTs) have compared the effectiveness of various adjuvant chemotherapy regimens for these patients [9–13]. However, clinical studies that evaluate the effects of adjuvant chemotherapy tend to have a selection bias toward younger patients.

A pooled analysis of RCTs from the US and several European countries reported that adjuvant chemotherapy increased survival and time to tumor recurrence in older patients with stage III colon cancer [14]. Two other retrospective population-based cohort studies have also produced similar results [15, 16]. Based on this evidence, Japanese treatment guidelines currently recommend adjuvant chemotherapy for older patients with stage III colon cancer [17]. However, treatment strategies for colon cancer in Japan involve the use of complete mesocolic excision (CME) with central vascular ligation as standard procedure, which differs from the approaches used in numerous other countries [17]. Furthermore, Japan has approved the use of several types of adjuvant chemotherapy regimens for stage III colon cancer patients that are not yet approved in other countries. In addition to fluorouracil plus leucovorin (5-FU/LV), capecitabine (CAPE), 5-FU/LV plus oxaliplatin (FOLFOX), and CAPE plus oxaliplatin (CAPEOX), adjuvant chemotherapy is also administered with uracil-tegafur plus leucovorin (UFT/LV) and tegafur/gimeracil/oteracil/potassium (S-1). S-1 plus oxaliplatin (SOX) has also been successfully used in a clinical trial [18]. Despite the availability of these options, patients aged ≥75 years with stage III colon cancer in Japan are undertreated with adjuvant chemotherapy [19]. Additionally, the effectiveness of adjuvant therapy for colon cancer in patients aged < 75 years has been investigated in many clinical trials and the benefits of patients aged ≥75 years remain unclear [11, 20, 21].

This study aimed to assess the effectiveness of postoperative adjuvant chemotherapy on survival benefit in stage III colon cancer patients stratified by age, and to characterize the types of adjuvant chemotherapy regimens used in a real-world setting in Japan.

## Methods

### Data and cohort construction

In this retrospective cohort study, we performed a record linkage of population-based cancer registry data and administrative data to analyze the relationship between patient mortality and clinical information that is unavailable in cancer registries. Data were obtained from a 5-year period spanning January 2010 to December 2014. The study protocol was approved by the Institutional Review Board of Osaka International Cancer Institute (Approval number: 1707105108).

Cancer registry data were obtained from the Osaka Cancer Registry, a population-based registry that compiles information on cancer diagnoses and outcomes in patients residing in Osaka Prefecture (the third most populous prefecture after Tokyo and Kanagawa). Data include patient sex, age at cancer diagnosis, use or non-use of curative resection and adjuvant chemotherapy for colon cancer, surgical approach (laparoscopic or open), vital status, and dates of death or the last follow-up to determine vital status. Tumor-specific data include primary location, degree of differentiation, date of cancer diagnosis, and Surveillance, Epidemiology, and End Results (SEER) summary stage [22].

The administrative data used in this study were produced under Japan’s Diagnosis Procedure Combination (DPC) per-diem payment system, which determines insurers’ reimbursements to acute care hospitals for the provision of healthcare services. These data are widely used for research in Japan [23]. DPC data comprise clinical summaries and detailed insurance claims, and include information on comorbidities, preoperative and postoperative activities of daily living (ADL), postoperative complications, and prescribed drugs. The data were obtained from 36 government-designated cancer care hospitals located throughout Osaka Prefecture.

The two data sources were linked at the patient level using each hospital’s patient identification number as a linkage key [24]. This record-linked database encompassed approximately 50% of all newly diagnosed cancer cases in Osaka Prefecture during the study period.

Colon cancer cases were identified using the relevant International Classification of Diseases for Oncology, Third Edition (ICD-O3) topographical codes. Candidate subjects comprised patients aged 18 years or older who had been diagnosed with colon cancer (C18.0, C18.2–C18.8, or C19.9) at any of the 36 cancer care hospitals and had been registered in the Osaka Cancer Registry between January 1, 2010 and December 31, 2014. We focused on patients who received a diagnosis of adenocarcinoma (ICD-O3 morphological codes: 8140, 8211, 8260, 8480, 8490, or 8510) with regional lymph node metastasis according to SEER staging criteria, and had undergone curative resection for colon cancer. Regional lymph node metastasis (T1–T4a, N1–N2, M0) in the SEER staging system was ascertained in stage III (T1–T4, N1–N2, M0) patients according to the TNM classification system (version 7) of the American Joint Committee on Cancer [25]. Patients were excluded from analysis if they had missing vital status data, or had died within 90 days from the cancer diagnosis.

The exposure of interest was the use of adjuvant chemotherapy, which was defined as the administration of chemotherapy within four months after surgical resection that was included in the initial cancer treatment plan. The following seven types of adjuvant chemotherapy were identified in the DPC data for analysis: 5-FU/LV, CAPE, UFT/LV, S-1, FOLFOX, CAPEOX, and SOX. The first three regimens are types of single-agent chemotherapy (monotherapy), and the remaining four regimens are types of double-agent chemotherapy (combined therapy).

The endpoint was overall survival (OS), which was calculated as the number of days from the date of cancer diagnosis until the date of all-cause death, loss to follow-up, or alive through May 2018.

Several demographic and clinical variables were obtained for analysis. These included sex, age at diagnosis (< 75 and ≥ 75 years), and degree of tumor differentiation (well-, moderately, and poorly differentiated). In addition, tumor localization was divided into two anatomical subsites: right colon (ICD-O3 topographical codes: C18.0 and C18.2–C18.5) and left colon (C18.6–C18.8 and C19.9). Body mass index was classified into four categories (< 18.5, 18.5–24.9, 25.0–29.9, and ≥ 30 kg/m^2^). The Charlson comorbidity index (CCI) was used to measure patients’ comorbidities on admission for radical surgery for colon cancer, [26, 27] and was classified into four categories based on CCI scores (0, 1, 2, or ≥ 3). The Barthel index was used to measure patients’ ADL on admission and discharge for radical surgery for colon cancer; this index uses a scale from 0 to 100, and was classified into five categories (0–20, 21–60, 61–90, 90–99, 100) for analysis [28]. The following postoperative complications were identified from the relevant data fields in the DPC file using International Classification of Diseases, Tenth Revision codes: surgical site infection (T79.3 or T81.4), peritonitis or peritoneal abscess (K65.x), pancreatic injury (K91.8), ileus (K56.x or K91.3), anastomotic stenosis (T81.8), sepsis (A40.x or A41.x), respiratory complication (pneumonia [J12.x–J18.x], postprocedural respiratory disorder [J95.x], or respiratory failure [J96.x]), pulmonary embolism (I26.x), cardiac event (acute coronary event [I21.x–I24.x] or heart failure [I50.x]), cerebral infarction or hemorrhage (I60.x–I64.x), acute renal failure (N17.x), and urinary tract infection (N10.x, N30.x, or N39.0) [29].

### Statistical analyses

The clinical factors and adjuvant chemotherapy regimens for each of the patient age groups (< 75 and ≥ 75 years) were analyzed using descriptive statistics. We examined frequencies for categorical variables and medians with interquartile range (IQR) for continuous variables. Univariate analyses were employed to compare the variables between the two patient age groups, in which categorical variables were compared with Fisher’s exact test and continuous variables were compared with the Mann-Whitney *U* test.

Survival analysis was performed using the Kaplan-Meier method, and survival estimates were compared using the log-rank test. The effect of adjuvant chemotherapy on OS was analyzed using Cox proportional hazards regression models for all-cause mortality with inverse probability weighting based on propensity scores to adjust for differences between the postoperative treatment groups (surgery with adjuvant chemotherapy and surgery alone) [30]. In order to determine the inverse probability of treatment weights, each patient’s propensity score was calculated using logistic regression to estimate the probability of receiving adjuvant chemotherapy conditional to his/her characteristics: sex, age at diagnosis, degree of tumor differentiation, radical surgery procedure, tumor location, body mass index, preoperative and postoperative Barthel index, CCI, and postoperative complications. We then assigned patients who underwent postoperative adjuvant chemotherapy a weight of 1÷(propensity score) and those who underwent surgery alone a weight of 1÷(1 − propensity score). We assessed covariate balance using absolute standardized differences; a difference of 10% or less was considered to indicate a well-balanced result [31]. Adjusted hazard ratios (HRs) and 95% confidence intervals (CIs) were estimated for each patient age group. Missing data were imputed using the multiple imputation method [32]. We also estimated adjusted HRs using complete data (without missing data imputation) as a sensitivity analysis.

All significance tests were 2-sided and *P* values < 0.05 were considered to be statistically significant. Statistical analyses were performed using STATA version 15.1 software (STATA Corporation, College Station, TX, USA).

The datasets used and/or analyzed during the current study are available from the corresponding author on reasonable request.

## Results

**Fig. 1:**
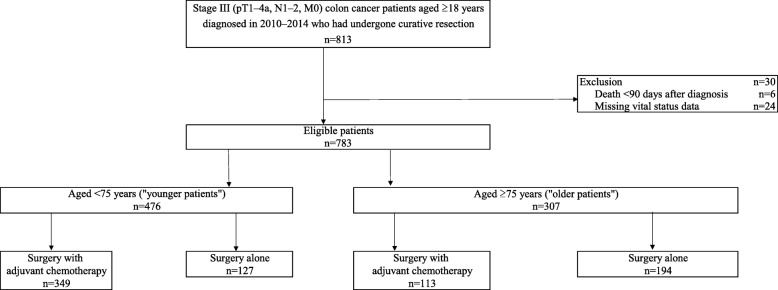
Overview of patient selection and use of adjuvant chemotherapy stratified by age

We identified 783 eligible patients aged 18 years or older who underwent curative resection and were diagnosed with stage III colon cancer between 2010 and 2014 (Fig. 1). Of these patients, 476 (60.8%) were aged < 75 years (younger patients), and 307 patients (39.2%) were aged ≥75 years (older patients). A total of 349 younger patients (73.3%) and 113 older patients (36.8%) received adjuvant chemotherapy after surgery. The median follow-up time was 1495 days (IQR: 1315–1979 days), and 192 patients (24.5%) died during follow-up.

The patient characteristics are summarized in Table 1. The median age was 66 years (IQR: 60–71 years) in the younger patients and 80 years (IQR: 76–83 years) in the older patients. When compared with older patients, a higher percentage of younger patients had tumors in the left colon (64.1% vs 48.0%; *P* = 0.001), no comorbidities (CCI = 0) (79.0% vs 57.9%; *P* < 0.001), and good preoperative (93.3% vs 77.9%; *P* < 0.001) and postoperative (95.0% vs 79.1%; *P* < 0.001) ADL (Barthel index = 100). In addition, a lower percentage of younger patients had postoperative complications (8.4% vs 12.8%; *P* = 0.022).

**Table 1:** Patient characteristics

	Total (*n* = 783)				< 75 years (*n* = 476)				≥75 years (*n* = 307)				*P* value
Male, n (%)	402	(	51.3	%)	250	(	54.1	%)	152	(	47.4	%)	0.42
Age in years, median (IQR)	72	[	65–78	]	66	[	60–71	]	80	[	76–83	]	< 0.001
Body mass index in kg/m^2^, n (%)													
< 18.5	88	(	11.2	%)	50	(	10.8	%)	38	(	11.8	%)	0.76
18.5–24.9	523	(	66.8	%)	321	(	69.5	%)	202	(	62.9	%)	
25.0–29.9	137	(	17.5	%)	85	(	18.4	%)	52	(	16.2	%)	
≥30	25	(	3.2	%)	17	(	3.7	%)	8	(	2.5	%)	
Unknown	10	(	1.3	%)	3	(	0.6	%)	7	(	2.2	%)	
Tumor differentiation, n (%)													
Well	188	(	24.0	%)	110	(	23.8	%)	78	(	24.3	%)	0.28
Moderate	499	(	63.7	%)	309	(	66.9	%)	190	(	59.2	%)	
Poor	29	(	3.7	%)	14	(	3.0	%)	15	(	4.7	%)	
Unknown	67	(	8.6	%)	43	(	9.3	%)	24	(	7.5	%)	
Tumor location, n (%)													
Right colon	331	(	42.3	%)	179	(	38.7	%)	152	(	47.4	%)	0.001
Left colon	450	(	57.5	%)	296	(	64.1	%)	154	(	48.0	%)	
Unknown	2	(	0.3	%)	1	(	0.2	%)	1	(	0.3	%)	
Charlson comorbidity index, n (%)													
0	551	(	70.4	%)	365	(	79.0	%)	186	(	57.9	%)	< 0.001
1	40	(	5.1	%)	20	(	4.3	%)	20	(	6.2	%)	
2	111	(	14.2	%)	48	(	10.4	%)	63	(	19.6	%)	
≥3	81	(	10.3	%)	43	(	9.3	%)	38	(	11.8	%)	
Preoperative Barthel index, n (%)													
0–20	19	(	2.4	%)	6	(	1.3	%)	13	(	4.0	%)	< 0.001
21–60	30	(	3.8	%)	9	(	1.9	%)	21	(	6.5	%)	
61–90	40	(	5.1	%)	24	(	5.2	%)	16	(	5.0	%)	
91–99	12	(	1.5	%)	6	(	1.3	%)	6	(	1.9	%)	
100	681	(	87.0	%)	431	(	93.3	%)	250	(	77.9	%)	
Unknown	1	(	0.1	%)	0	(	0.0	%)	1	(	0.3	%)	
Surgical approach, n (%)													
Open	250	(	31.9	%)	147	(	31.8	%)	103	(	32.1	%)	0.43
Laparoscopic	533	(	68.1	%)	329	(	71.2	%)	204	(	63.6	%)	
Postoperative complications, n (%)													
Yes	80	(	10.2	%)	39	(	8.4	%)	41	(	12.8	%)	0.022
Postoperative Barthel index, n (%)													
0–20	9	(	1.1	%)	2	(	0.4	%)	7	(	2.2	%)	< 0.001
21–60	28	(	3.6	%)	7	(	1.5	%)	21	(	6.5	%)	
61–90	39	(	5.0	%)	21	(	4.5	%)	18	(	5.6	%)	
91–99	13	(	1.7	%)	7	(	1.5	%)	6	(	1.9	%)	
100	693	(	88.5	%)	439	(	95.0	%)	254	(	79.1	%)	
Unknown	1	(	0.1	%)	0	(	0.0	%)	1	(	0.3	%)	
Adjuvant chemotherapy													
Yes	462	(	59.0	%)	349	(	73.3	%)	113	(	36.8	%)	< 0.001
IQR, interquartile range.													

Table 2 shows the distribution of adjuvant chemotherapy regimens in all patients and the two age groups. Among the 462 patients who received adjuvant chemotherapy, 298 patients (64.5%) received monotherapy and 164 patients (35.5%) received combined therapy. UFT/LV was the most commonly used regimen (*n* = 139 [30.1%]), especially in older patients (*n* = 47 [41.6%]). The percentage of younger patients who received combined therapy was approximately twice that of older patients (40.7% vs 19.5%).

**Table 2:** Distribution of adjuvant chemotherapy regimens among patients

	Total (*n*=462)				<75 years (*n*=349)				≥75 years (*n*=113)				*P* value
	n		%		n		%		n		%		
Single-agent chemotherapy	298	(	64.5	%)	207	(	59.3	%)	91	(	80.5	%)	<0.001
5-FU/LV	13	(	2.8	%)	11	(	3.2	%)	2	(	1.8	%)	
CAPE	100	(	21.6	%)	75	(	21.5	%)	25	(	22.1	%)	
UFT/LV	139	(	30.1	%)	92	(	26.4	%)	47	(	41.6	%)	
S-1	46	(	10.0	%)	29	(	8.3	%)	17	(	15.0	%)	
Double-agent chemotherapy	164	(	35.5	%)	142	(	40.7	%)	22	(	19.5	%)	
FOLFOX	35	(	7.6	%)	26	(	7.4	%)	9	(	8.0	%)	
CAPEOX	126	(	27.3	%)	114	(	32.7	%)	12	(	10.6	%)	
SOX	3	(	0.6	%)	2	(	0.6	%)	1	(	0.9	%)	

5-FU/LV, fluorouracil plus leucovorin; CAPE, capecitabine; UFT/LV, uracil-tegafur plus leucovorin; S-1, tegafur/gimeracil/oteracil/potassium; FOLFOX, 5-FU/LV plus oxaliplatin; CAPEOX, CAPE plus oxaliplatin; SOX, S-1 plus oxaliplatin

**Fig. 2:**
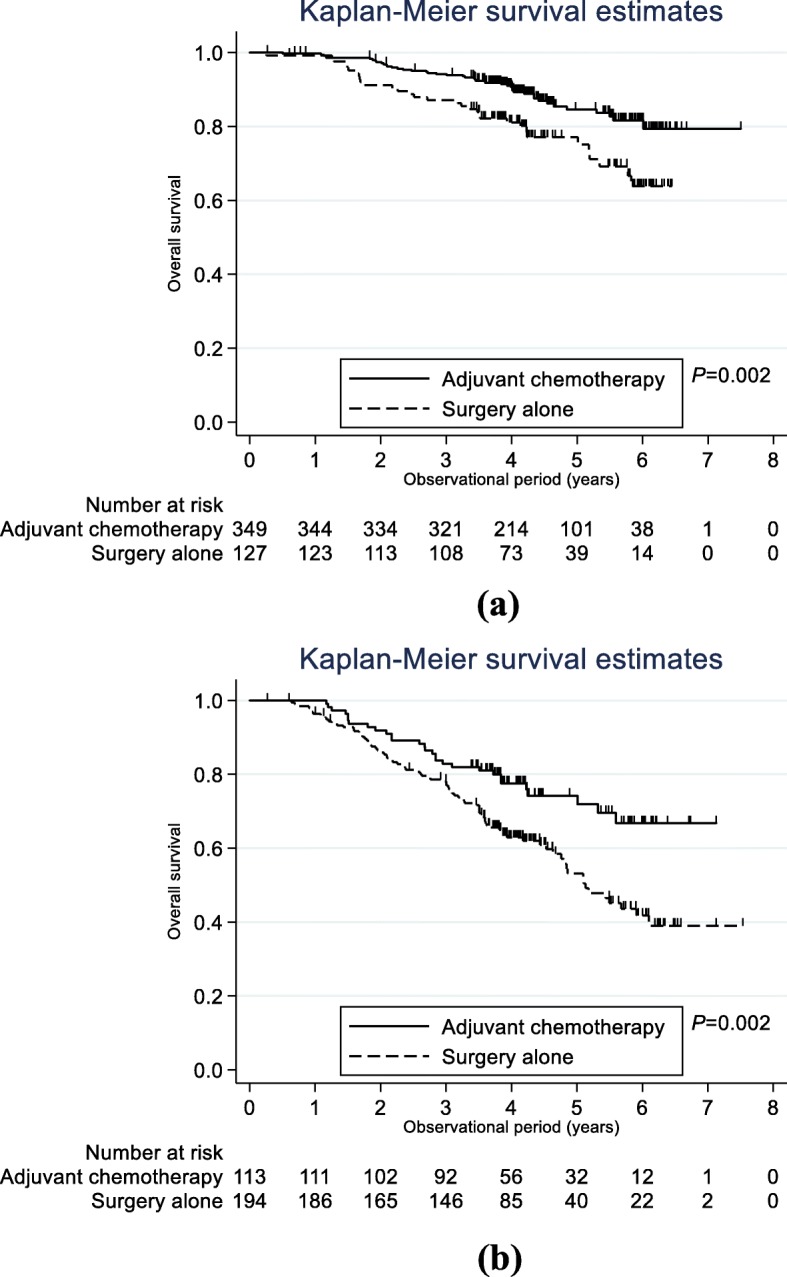
Overall survival according to the use or non-use of adjuvant chemotherapy in (**a**) patients aged < 75 and (**b**) patients aged ≥75 years

OS analysis was performed using the Kaplan-Meier method (Fig. 2). For younger patients (Fig. 2a), the 3-year estimated OS for surgery with adjuvant chemotherapy and surgery alone was 94.2% (95% CI: 91.1–96.2%) and 87.1% (95% CI: 79.8–91.9%), respectively (*P* = 0.002). Among these patients, the 5-year estimated OS for surgery with adjuvant chemotherapy and surgery alone was 84.6% (95% CI: 79.1–88.7%) and 77.1% (95% CI: 67.9–84.0%), respectively (*P* = 0.002). For older patients (Fig. 2b), the 3-year estimated OS for surgery with adjuvant chemotherapy and surgery alone was 82.9% (95% CI: 74.5–88.7%) and 78.0% (95% CI: 71.5–83.3%), respectively (*P* = 0.002). Among these patients, the 5-year estimated OS for surgery with adjuvant chemotherapy and surgery alone was 74.2% (95% CI: 64.0–81.9%) and 53.1% (95% CI: 44.1–61.3%), respectively (*P* = 0.002).

The effect of adjuvant chemotherapy on survival benefit was estimated using Cox proportional hazards regression models for all-cause mortality with inverse probability weighting of propensity scores (Table 3). The covariates were well-balanced in the propensity-weighted cohort, with all standardized differences below 10% (data not shown). For younger patients, the adjusted HR of surgery with adjuvant chemotherapy compared with surgery alone was 0.56 (95% CI: 0.33–0.94, *P* = 0.027). For older patients, the adjusted HR of surgery with adjuvant chemotherapy compared with surgery alone was 1.07 (95% CI: 0.66–1.74, *P* = 0.78). The sensitivity analysis with complete data similarly showed different effects of adjuvant chemotherapy between these groups: the adjusted HR of surgery with adjuvant chemotherapy was 0.55 (95% CI: 0.33–0.94, *P* = 0.028) in younger patients and 1.13 (95% CI: 0.71–1.81, *P* = 0.60) in older patients.

**Table 3:** Adjusted hazard ratios of all-cause mortality using Cox proportional hazards regression models with inverse probability weighting of the propensity score

	Multiple imputation of missing data			Complete data analysis		
	Adjusted HR	95% CI	*P* value	Adjusted HR	95% CI	*P* value
< 75 years						
Surgery alone	(reference)			(reference)		
Surgery with adjuvant chemotherapy	0.56	(0.33–0.94)	0.027	0.55	(0.33–0.94)	0.028
≥75 years						
Surgery alone	(reference)			(reference)		
Surgery with adjuvant chemotherapy	1.07	(0.66–1.74)	0.78	1.13	(0.71–1.81)	0.60

Adjusted for sex, age at diagnosis, degree of tumor differentiation, radical surgery procedure, tumor location, body mass index, preoperative and postoperative Barthel index, comorbidities, and postoperative complications

CI, confidence interval; HR, hazard ratio

## Discussion

To our knowledge, this represents one of the largest Japanese real-world studies to date that evaluates the effectiveness of adjuvant chemotherapy after surgical resection in older patients with stage III colon cancer that accounts for potential confounding factors [19]. The main study findings are as follows: although adjuvant chemotherapy demonstrated a significant association with improved survival among younger patients aged < 75 years, no such association was observed among older patients aged ≥75 years. Next, the utilization rate of adjuvant chemotherapy was 59% among all patients; of these, 64.5% received monotherapy and 35.5% received combined therapy. UFT/LV was the most commonly used regimen. In addition, the 5-year OS for older patients with stage III colon cancer for surgery with adjuvant chemotherapy and surgery alone was 74.2 and 53.1%, respectively.

The lack of a significant association between adjuvant chemotherapy and longer survival in older patients could be due to a variety of factors, such as patient selection, the relatively small sample size, the types of adjuvant chemotherapy used, and unmeasured confounding. While younger patients who received adjuvant chemotherapy had longer survival times than those who underwent surgery alone, the benefits of adjuvant chemotherapy appeared to be smaller in older patients in a real-world setting. Additionally, the log-rank test indicated that adjuvant chemotherapy was associated with longer survival in older patients, but the Cox proportional hazard model showed no statistical significance and an HR > 1 for mortality. We posit that this small increase in HR may have been influenced by the presence of unmeasured confounding factors, such as lymph node metastasis (an important prognostic factor for stage III colon cancer), as this information was not included in the study data. Furthermore, other unmeasured confounders such as socioeconomic status (which is a risk factor for poor survival among cancer patients and is associated with a lower frequency of adjuvant chemotherapy use) are likely to create a bias in favor of adjuvant chemotherapy. In this way, the observed effect of adjuvant chemotherapy for older patients with stage III colon cancer may be smaller than expected. Because this study showed no significant difference in the effect of adjuvant chemotherapy in older patients, further analyses are needed to verify these findings and to explore the possible underlying causes.

The standard surgical treatment of colon cancer in Japan involves CME with central vascular ligation, which differs from that of many European countries and the US. CME is based on the principles of total mesorectal excision, where the entire embryological mesocolon (from the viscera to the parietal planes) is excised horizontally. This procedure has been reported to improve long-term outcomes in colon cancer without major surgical risks [33, 34]. On the other hand, the effects of central vascular ligation are more controversial [35]. In Japan, this technique has been employed as a standard surgical procedure for colon cancer for the several decades. In contrast, countries in Europe and the US proposed CME as a standardized surgical technique in 2009, but central vascular ligation remains a non-standardized procedure. These differences in surgical procedures should be kept in mind when interpreting and comparing the findings of studies on the effects of adjuvant chemotherapy.

Previous studies have reported similar effects of adjuvant chemotherapy for both younger and older patients [14–16]. However, these studies have included few, if any, Asian patients. Other articles have reported that the prognosis of Asian people with colon cancer is better than non-Asians [36, 37]. This racial difference may affect the age-dependent benefits of adjuvant chemotherapy. A Japanese study previously examined the effectiveness of adjuvant chemotherapy in older patients using data from the Japanese Study Group for Postoperative Follow-up of Colorectal Cancer, which comprises colorectal cancer treatment experts in Japan; that study concluded that adjuvant chemotherapy is effective in improving OS in older patients with stage III colon cancer [19]. However, their study was conducted using data from 2004 to 2006, and only accounted for a few confounding factors. Furthermore, the popularization of laparoscopic surgery during that period may have helped to reduce postoperative complications. These factors may have contributed to that study’s conclusion that adjuvant chemotherapy is effective for older patients with stage III colon cancer.

The utilization rate of adjuvant chemotherapy was 59% among all patients (monotherapy: 64.5%, combined therapy: 35.5%). A previous study reported that 44.8% of cancer patients in Japan received adjuvant chemotherapy [38]. In this way, the proportion of Japanese patients who receive adjuvant chemotherapy (especially combined therapy) is generally lower than that reported in other countries [39, 40]. This relatively infrequent use of adjuvant chemotherapy may affect its perceived benefits in older Japanese patients with stage III colon cancer. In addition, the lack of adjuvant chemotherapy (especially combined therapy) may contribute to poorer prognoses in these patients.

UFT/LV was the most common type of adjuvant chemotherapy administered in our subjects, particularly in the older patients. In addition, 15% of older patients received S-1. In contrast, other countries have reported CAPE to be the most popular oral 5-FU prodrug [41, 42]. In Japan, however, oral 5-FU derivatives are preferred because of their convenience, leading to the development of several oral 5-FU derivatives with different properties, such as UFT/LV and S-1. UFT/LV is commonly used for stage III colon cancer patients in Japan because its non-inferiority to intravenous 5-FU/LV has been confirmed by RCTs [11, 43]. Another trial determined that S-1 for stage III colon cancer is non-inferior to UFT/LV [12]. However, it remains unclear as to which oral 5-FU is the most effective, especially in older patients [13].

These findings should be interpreted with consideration to several limitations. Firstly, we could not investigate the severity of lymph node metastasis—which is an important prognostic factor for stage III colon cancer—as this information was not included in the study data. Secondly, this study did not include any patients with stage T4b cancer (involving the direct invasion of adjacent organs), which is also an important negative prognostic factor. The lack of such patients could result in selection bias. Thirdly, we could not investigate the cause of death, presence or absence of recurrence, adverse events of adjuvant chemotherapy, or the quality of surgery due to data limitations. Furthermore, the data lacked detailed clinical information (such as lymph node metastasis or the reasons for each patient’s treatment plan), which is an intrinsic limitation of using cancer registry data. As a result, there may still be unmeasured confounding factors in the analysis. Next, the sample size of this study may still be relatively small despite being conducted in a real-world setting in one of Japan’s largest prefectures. This may also contribute to selection bias. The inclusion of other types of registry data may enable further investigations into these limitations. Finally, we were unable to examine if the effects of adjuvant chemotherapy were attributable to single-agent chemotherapy or double-agent chemotherapy because the study’s primary objective was to determine whether adjuvant chemotherapy was associated with improved survival irrespective of regimen type. Further studies are needed to identify the optimal regimen for stage III colon cancer patients in the real-world setting.

This study is, to the best of our knowledge, the first Japanese real-world cohort study that uses a combination of cancer registry data and administrative data to examine the survival benefit of adjuvant chemotherapy in stage III colon cancer patients. In clinical practice, clinicians often struggle with treatment strategies for subpopulations that are generally not included in RCTs, such as older patients. The real-world setting of this study is useful for the resolution of this issue. Additionally, the combination of two different datasets helped to overcome the limitations of each data source. Because the Japanese treatment strategies for stage III colon cancer patients have demonstrated effectiveness in several RCTs, [11–13, 18, 43] our findings are potentially generalizable to hospitals that employ these strategies.

## Conclusions

Adjuvant chemotherapy did not demonstrate any significant effects on survival benefit in older patients with stage III colon cancer under current utilization practices. These real-world findings may have applications in informing the treatment strategies of older patients, who are frequently not included in the population of RCTs.

## Data Availability

The datasets used and/or analyzed during the current study are available from the corresponding author on reasonable request.

## Abbreviations

5-FU/LV: Fluorouracil plus leucovorin
ADL: Activities of daily living
CAPE: Capecitabine
CAPEOX: CAPE plus oxaliplatin
CCI: Charlson comorbidity index
CI: Confidence interval
CME: Complete mesocolic excision
DPC: Diagnosis Procedure Combination
FOLFOX: 5-FU/LV plus oxaliplatin
HR: Hazard ratio
ICD-O3: International Classification of Diseases for Oncology, Third Edition
IQR: Interquartile range
OS: Overall survival
RCT: Randomized controlled trial
S-1: Oral fluoropyrimidine
SEER: Surveillance, Epidemiology, and End Results
SOX: S-1 plus oxaliplatin
UFT/LV: Uracil-tegafur plus leucovorin
